# ﻿Review of the genus *Xenicotela* Bates, 1884 (Cerambycidae, Lamiinae, Lamiini)

**DOI:** 10.3897/zookeys.1183.112490

**Published:** 2023-11-07

**Authors:** Guanglin Xie, Maxwell V. L. Barclay, Wenkai Wang

**Affiliations:** 1 Institute of Entomology, College of Agriculture, Yangtze University, Jingzhou, Hubei, 434025, China; 2 Department of Life Sciences, Natural History Museum, London, SW7 5BD, UK; 3 Hubei Engineering Research Center for Pest Forewarning and Management, Yangtze University, Jingzhou, Hubei, 434025, China

**Keywords:** Coleoptera, Cerambycidae, identification key, new species, new combination, taxonomy

## Abstract

The species of the genus *Xenicotela* Bates, 1884 are reviewed. One new species, *Xenicotelamucheni***sp. nov.**, is described from Yunnan, China. *Monochamusbinigricollis* Breuning, 1965 and *Monochamusvilliersi* Breuning, 1960 are transferred to *Xenicotela* as follows: *Xenicotelavilliersi* (Breuning, 1960) **comb. nov.** and *Xenicotelabinigricollis* (Breuning, 1965) **comb. nov.***Xenicoteladistincta* (Gahan, 1888) is newly reported from Myanmar and *Xenicotelabinigricollis* is excluded from the fauna of China. All species are redescribed and illustrated. A key to the known *Xenicotela* species is provided.

## ﻿Introduction

Bates (1884) established the genus *Xenicotela* for *Xenicotelafuscula* Bates, 1884 [currently considered a synonym of *Xenicotelapardalina* (Bates, 1884)] from Higo, Japan. Recently, Xie et al. (2022) reviewed the Chinese species of the genus, increasing the number of species to five, which are known from Japan, South Korea, China, Vietnam, Laos, Nepal and India.

The present paper is a further study. The genus is reviewed, a new species is described, two *Monochamus* Dejean, 1821 species are transferred to *Xenicotela*, new photographs of the types of *Monohammusdistinctus* Gahan, 1888 and *Nephelotustonkineus* Pic, 1926 are provided and a key to the known species is given.

## ﻿Material and methods

Specimens from the following institutional or private collections were examined and/or photographed in this study:

**BPBM**Bernice Pauahi Bishop Museum, Honolulu, USA;

**CQNU**Chongqing Normal University, Chongqing, China;

**GZNULS**School of Life Sciences, Guizhou Normal University, Guiyang, China;

**IZAS**Institute of Zoology, Chinese Academy of Sciences, Beijing, China;

**LGBC** Collection of Larry G. Bezark, Sacramento, California, USA;

**MCC** Collection of Mu Chen, Shanghai, China;

**MNHN**Muséum National d’Histoire Naturelle, Paris, France;

**NHMUK**Natural History Museum, London, UK;

**NOC** Collection of Nobuo Ohbayashi, Miura, Japan;

**SWU** Southwest University, Chongqing, China;

**YZU** Yangtze University, Jingzhou, China.

The male genitalia of the new species were dissected and soaked in glycerine in a centrifuge tube. The genitalia were prepared by first soaking the whole beetle in boiling water for several minutes, then opening the abdomen from the apex along the dorsopleural margin. The genitalia were then removed with fine forceps and ophthalmic scissors, and later cleared in 10% KOH at 80–100 °C for several minutes.

All photographs of the habitus were taken using a Canon 7D Mark II digital camera equipped with a Canon EF 100 mm f/2.8L IS USM, while images of genitalia were taken with a Leica DFC450 digital camera mounted on a Leica M205A microscope. Images of genitalia were taken by keeping them in glycerine. All images were edited using Adobe Photoshop 2020 release.

## ﻿Taxonomy

### ﻿Genus Xenicotela

Taxon classificationAnimaliaColeopteraCerambycidae

Bates, 1884

9CA76342-C98E-58CF-98D8-E7EB79A6748E

Xenicotela Bates, 1884: 242; Matsushita 1933: 346; Breuning 1944: 372; Gressitt 1951: 381; Breuning 1961: 353; Rondon and Breuning 1970: 458; Makihara 2007: 602; Hubweber et al. 2010: 288; Lin and Tavakilian 2019: 324; Xie et al. 2022: 145.

#### Type species.

*Xenicotelafuscula* Bates, 1884 [= *Xenicotelapardalis* (Bates, 1884)].

#### Diagnosis.

Small-bodied; body length usually less than 20 mm. Eyes coarsely faceted. Antennae long and slender, usually more than twice body length in male and about twice body length in female; antennomeres III–XI basally and apically annulated with light-coloured pubescence, basal antennomeres distinctly fringed with sparse setae ventrally; scape short, distinctly constricted before the apex, with a narrow and completely closed cicatrix at apex; antennomere III distinctly longer than fourth, about 2.0 times as long as scape. Pronotum transverse, lateral spine short, tapered. Elytra elongate, with subparallel sides, apices rounded. Procoxal cavities closed posteriorly. Mesosternal process not tuberculate, mesocoxal cavities open at sides. Legs moderately long, femora clavate, mesotibiae without grooves near external apex, claws widely divergent.

#### Distribution.

Japan, South Korea, China, Vietnam, Laos, Myanmar (new country record), Nepal, India.

#### Comments.

The genus was often confused with *Monochamus* (Bates 1884; Gahan 1888; Gressitt 1942; Breuning 1960, 1965), probably due to the small body size, which makes it difficult to observe their differences. In fact, it can be easy distinguished from *Monochamus* by the mesotibiae lacking grooves near the apex.

### ﻿ Xenicotela pardalina

Taxon classificationAnimaliaColeopteraCerambycidae

(Bates, 1884)

80C93C47-853E-552F-82A6-3591F6457388

Figs 1 , 2

Monochamus pardalinus Bates, 1884: 239; Aurivillius 1922: 87; Matsushita 1933: 325. Type locality: Yuyama, Honshu, Japan.
Xenicotela pardalina : Breuning 1944: 373; Breuning 1961: 353; Makihara 2007: 602; Hubweber et al. 2010: 288.
Xenicotela fuscula Bates, 1884: 242; Aurivillius 1922: 216; Matsushita 1933: 346; Cho et al. 1963: 3. Type locality: Higo, Kyushu, Japan.

#### Type material examined.

**Figure 1.**
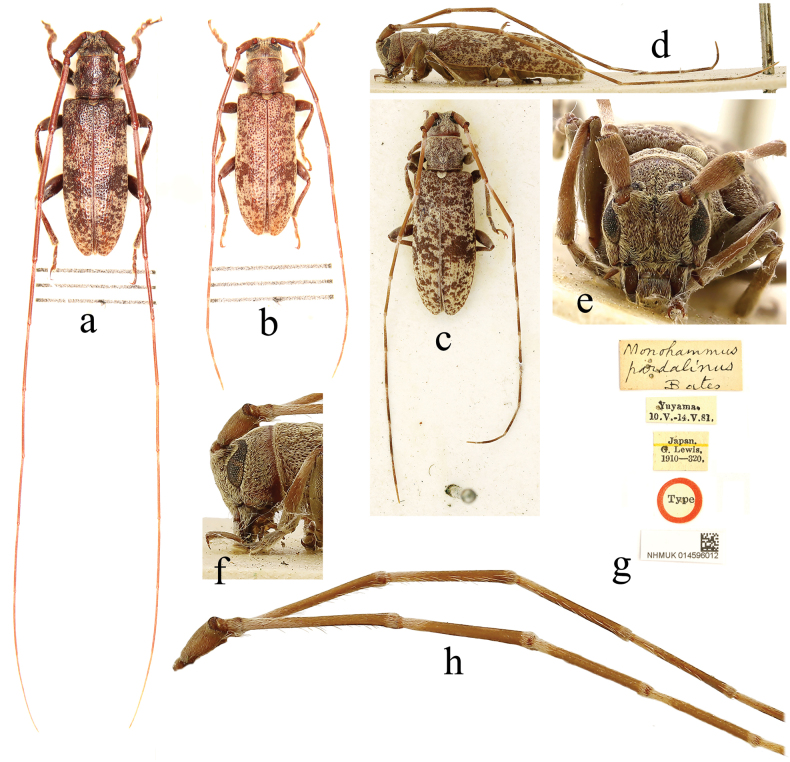
*Xenicotelapardalina* (Bates, 1884) **a** male, from Tottori Pref. (Mt. Daisen), Japan **b** female, from Tokushima Oref. (Dosu‐toge), Japan **c–h** holotype of *Monohammuspardalinus* Bates, 1884, female **a–c** dorsal view **d** lateral view **e** frontal view **f** head in lateral view **g** labels **h** antennomeres.

**Figure 2.**
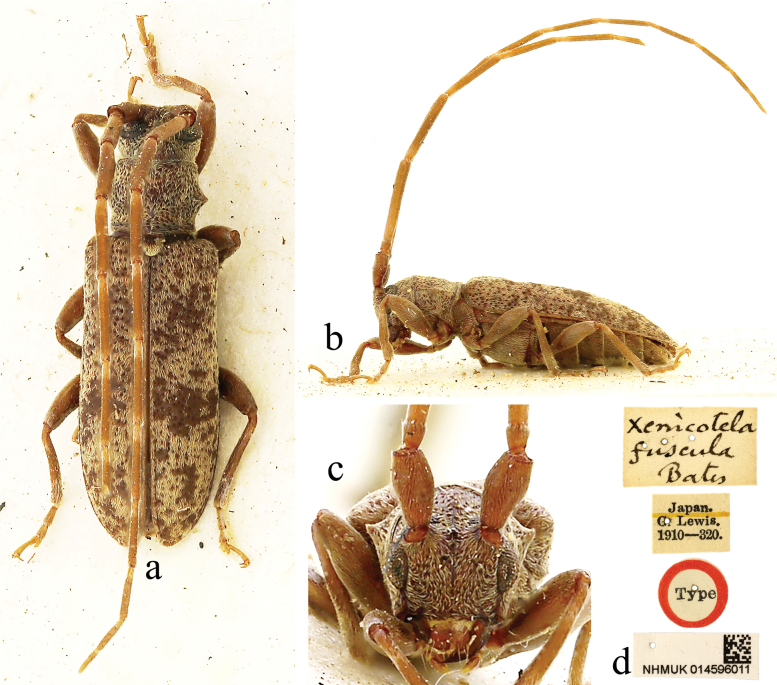
Holotype of *Xenicotelafuscula* Bates, 1884, female **a** dorsal view **b** lateral view **c** frontal view **d** labels.

***Holotype*** of *Monohammuspardalinus*, female (NHMUK); label details are shown in Fig. 1g. ***Holotype*** of *Xenicotelafuscula*, female (NHMUK); label details are shown in Fig. 2d.

#### Other material examined.

One male, Japan: Honshu, Tottori Pref., Mt. Daisen, July 22–23, 1974, coll. Y. Notsu (NOC); One female, Japan: Shikoku, Tokushima Oref., Dosu-toge, July 29, 1973, coll. M. Sakai (NOC).

#### Redescription.

**Female.** Body length 7.0–13.0 mm, humeral width 2.0–3.5 mm. Body reddish brown to blackish brown, clothed with greyish-yellow pubescence forming mottling on dorsal surface. Antennae slightly lighter in colour, basal four to six segments fringed with rather sparse setae ventrally, base and extreme apex of antennomeres III–X, base and apex of antennomere XI annulated with greyish-white pubescence. Elytra clothed with rather uneven pubescence forming irregular light patches interspersed with dark patches formed by ground colour of elytra, with a vague, incomplete, dark transverse band behind middle. Underside clothed with fairly even pubescence.

Head finely punctate; frons transverse, with a smooth longitudinal median sulcus extending to occiput; eyes coarsely faceted, lower lobe longer than gena. Antennae slender, about 2.0 times as long as body, with the apex of the sixth segment surpassing elytral apex; scape robust and short, base thin, apex distinctly constricted before cicatrix; antennomere III distinctly longer than antennomere IV, about 2.5 times as long as scape; antennomeres III–X slightly thickened at extreme apex. Pronotum transverse, lateral spine small and short, coniform; surface punctured similarly to head, disc slightly uneven. Scutellum lingulate. Elytra elongate, about 2.5 times as long as width across humeri; sides slightly expanded outwards after basal third, then evenly arched and narrowed backwards; apices rounded; surface punctures coarser and sparser than those on head and pronotum, sparser and even finer towards the apex; disc slightly depressed on the basal third. Legs moderately long, femora slightly clavate, claws divaricate.

**Male.** Similar to female, antennae thicker and longer, about 2.5–3.0 times as long as body, with the apex of the fifth segment or the base of the sixth segment exceeding the elytral apex.

#### Distribution.

Japan (Hokkaido, Honshu, Sado, Oki, Shikoku, Kyushu, Tanegashima, Yakushima), South Korea (Seoraksan).

#### Comments.

Bates (1884) described this species twice in the same publication, as *M.pardalinus* and *X.fuscula*, based on specimens from different localities. Breuning (1944) transferred *M.pardalinus* into the genus *Xenicotela* and proposed *X.fuscula* as its synonym. The holotype of *M.pardalinus* is larger in size than the holotype of *X.fuscula*, with longer antennae with the basal six antennomeres fringed with setae, the antennae appear darker in colour and the pubescent rings on the antennomeres look more obvious, while in the holotype of *X.fuscula*, only the basal four segments of the antennae are fringed with sparse setae, the fifth segment is only fringed with one or two setae and the pubescent rings on the antennomeres appear less distinct than those of the former specimen. These characters made them look like different species. Bates (1884) considered both holotypes to be males. In fact, they are females, according to the antennae / body length ratio (about 2: 1, in males 2.5–3: 1). Tavakilian and Chevillotte (2023) stated that types of both *X.pardalinus* and *X.fuscula* are conserved in the MNHN. This is not correct, as Bates described them from the collection of George Lewis (1839–1926), which is in the NHMUK.

### ﻿ Xenicotela distincta

Taxon classificationAnimaliaColeopteraCerambycidae

(Gahan, 1888)

3202F302-CD34-591F-B1D0-7CA102EE2B12

Figs 3 , 4 , 5

Monohammus distinctus Gahan, 1888: 392; Aurivillius 1922: 95. Type locality: Assam, India.
Xenicotela distincta : Breuning 1944: 373; Gressitt 1951: 382; Breuning 1961: 354; Rondon and Breuning 1970: 458; Hubweber et al. 2010: 288; Weigel et al. 2013: 288; Kariyanna et al. 2017: 253; Lin and Tavakilian 2019: 324; Xie et al. 2022: 147.
Nephelotus 4-maculatus Pic, 1925: 16. Type locality: Tonkin, Vietnam.
Nephelotus tonkineus Pic, 1926: 143. Type locality: Tonkin (Hoa Binh), Vietnam.
Xenicotela distincta m. *tonkinensis* Breuning, 1944: 373.
Monochamus binigricollis : Wang 1998: 599, misidentification.

#### Type material examined.

***Holotype*** of *Monohammusdistinctus*, male (NHMUK); label details are shown in Fig. 4e. ***Syntypes*** of *Nephelotustonkineus*, one male and one female (MNHN); label details are shown in Fig. 5e, j.

#### Other material examined.

One female, ‘Burmah’ (NHMUK); one female, India: Assam (NHMUK); One male, China: Yunnan Province, Cangyuan County, Daheishan, alt. 2400 m, May 15, 1980, coll. Kaiquan Li (SWU); One female, China: Guizhou Province, Ziyun County, Nazuo Village, June 8, 2019, coll. Shulin Yang (GZNULS); one female, China: Yunnan Province, Xishuangbanna Prefecture, Danuoyou, May 29, 2008, coll. Meiying Lin (IZAS); one male, China: Yunnan Province, Jiangcheng County, Qushui Township, alt. 564 m, 22°37'1"N, 102°9'49"E, June 8, 2019, coll. Lanbin Xiang (YZU).

#### Distribution.

China (Yunnan, Guizhou), India (Assam, Sikkim), Vietnam (Tonkin), Nepal, Laos, Myanmar (new country record).

#### Remarks.

**Figure 3.**
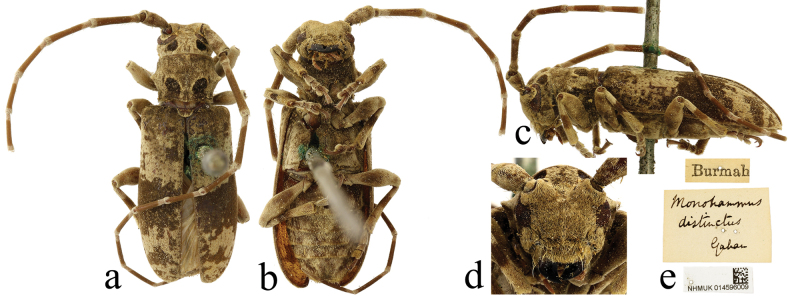
*Xenicoteladistincta* (Gahan, 1888), specimen from Myanmar **a** dorsal view **b** ventral view **c** lateral view **d** frontal view **e** labels.

One female specimen of this species labelled ‘Burmah’ (old spelling of Burma, i.e., Myanmar) was found in NHMUK (Fig. 3). This represents a new country record. A redescription and other comments about this species are provided by Xie et al. (2022).

**Figure 4.**
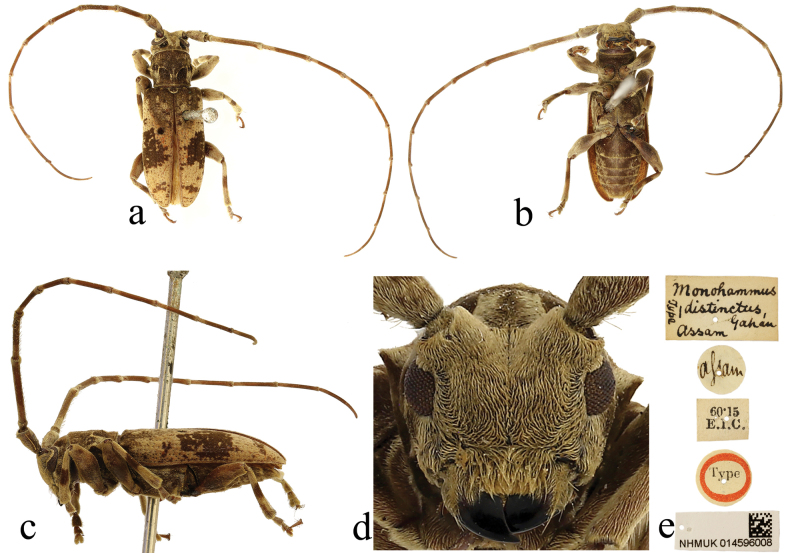
Holotype of *Monohammusdistinctus* Gahan, 1888 **a** dorsal view **b** ventral view **c** lateral view **d** frontal view **e** labels.

**Figure 5.**
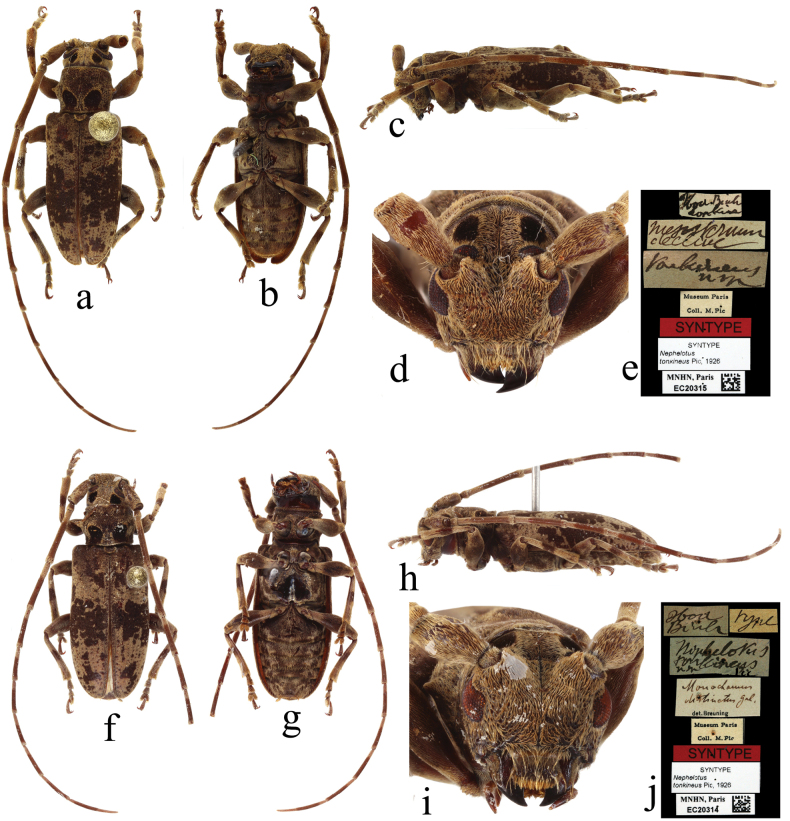
Syntypes of *Nephelotustonkineus* Pic, 1926 **a–e** male **f–j** female. **a, f** dorsal view **b, g** ventral view **c, h** lateral view **d, i** frontal view **e, j** labels.

### ﻿ Xenicotela bimaculata

Taxon classificationAnimaliaColeopteraCerambycidae

(Pic, 1925)

D61B6680-E195-5085-8962-436D94930FEE

Fig. 6

Nephelotus bimaculatus Pic, 1925: 16. Type locality: Tonkin (Hoa Binh), Vietnam.
Xenicotela bimaculatus : Breuning 1944: 373; Breuning 1961: 354.

#### Type material examined.

**Figure 6.**
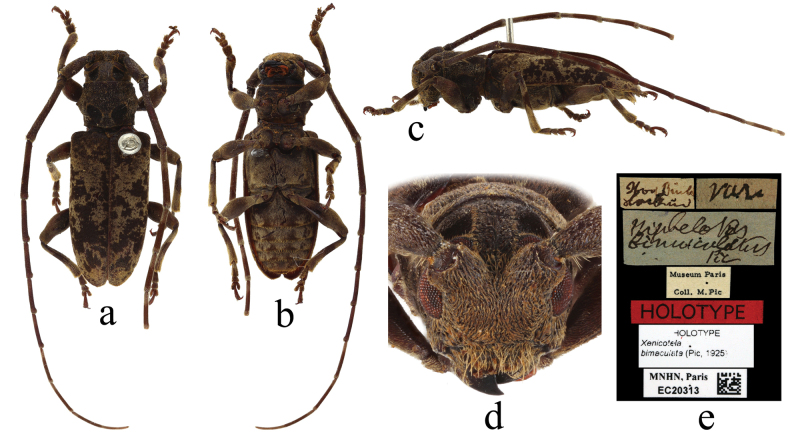
Holotype of *Nephelotusbimaculatus* Pic, 1925 **a** dorsal view **b** ventral view **c** lateral view **d** frontal view **e** labels.

***Holotype***, male (MNHN); label details are shown in Fig. 6e.

#### Redescription.

**Male.** Body length 13.0 mm, humeral width 4.0 mm. Body mostly blackish brown, clothed with yellowish and dark-brown pubescence. Frons clothed with uniform yellowish pubescence, decorated with a black velvet patch behind each upper eye lobe. Antennae reddish brown, basal five segments fringed with sparse setae ventrally, base and extreme apex of antennomeres III–X, base and apical half of antennomere XI annulated with spare greyish-yellow pubescence. Pronotum decorated with a black velvet patch edged with greyish-yellow to yellow pubescent border on each side of basal half. Scutellum clothed with yellowish pubescence at edges. Elytra dark brown, clothed with yellowish and dark-brown pubescence forming a mixture of irregular dark and light markings. Underside mostly clothed with greyish-yellow pubescence, ventrites fringed with short yellowish setae at posterior edge and with two dark-brown spots on each side forming two incomplete longitudinal stripes. Legs mostly reddish brown; femurs and tibiae decorated with a dark-brown median annulation.

Head finely punctate, frons transverse, lower eye lobes shorter than genae. Antennae long, about 2.0 times as long as body, scape robust, distinctly constricted before cicatrix; antennomere III a little longer than antennomere IV, about 2.0 times as long as scape; antennomeres III–X slightly protruding inwards at apex. Pronotum transverse; lateral spine short and blunt, coniform; disc punctured similarly to head. Scutellum lingulate. Elytra elongate, about 2.0 times as long as width across humeri, with subparallel sides and rounded apices; surface punctures similar to those of pronotum, gradually inconspicuous towards apex. Legs moderately long, femora slightly clavate, claws divaricate.

**Female.** Unknown.

#### Distribution.

Vietnam (Tonkin).

#### Comments.

This species is very similar to *X.distincta*, differing mainly in the elytral pattern. The irregular dark patches on the middle of each elytron are not connected into a large transverse band, while *X.distincta* shows a clear broad transverse middle dark band on each elytron. In addition, this species has the scutellum with the wider lighter-coloured pubescent border. The taxonomic status of this species needs to be further confirmed based on additional material.

### ﻿ Xenicotela convexicollis

Taxon classificationAnimaliaColeopteraCerambycidae

(Gressitt, 1942)

C3317FE0-B829-5724-A0A3-65A5780EC66B

Fig. 7a–f

Monochamus convexicollis Gressitt, 1942: 83; Gressitt 1951: 393; Breuning 1961: 370; Chou 2004: 296; Hubweber et al. 2010: 282; Lin and Tavakilian 2019: 310. Type locality: Zhejiang (Tianmushan), China.
Xenicotela convexicollis : Xie et al. 2022: 149.

#### Type material examined.

**Figure 7.**
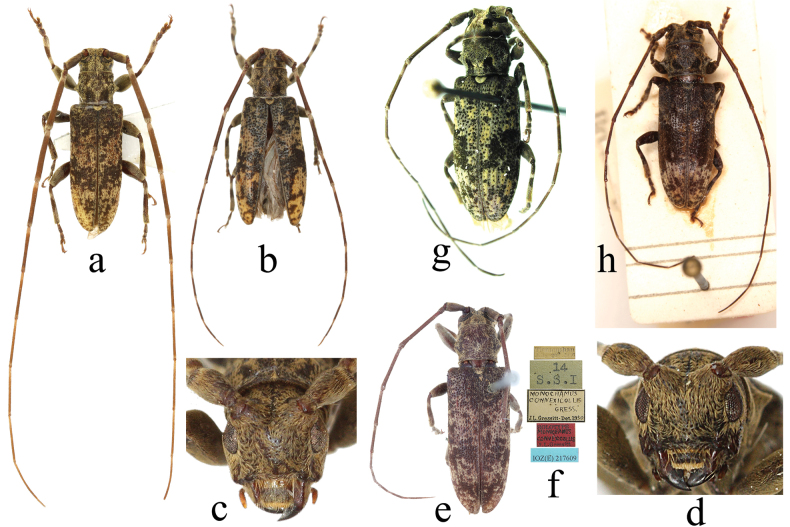
*Xenicotela* spp. **a–f***Xenicotelaconvexicollis* (Gressitt, 1942) **g–h***Xenicotelabinigricollis* (Breuning, 1965) comb. nov. **a, c** male, from Zhejiang (Tianmushan), China **b, d** female, from Zhejiang (Tianmushan), China **e, f** holotype (female) and labels **g** female, from Bac Kạn (Ba Be national park), Vietnam **h** holotype, female.

***Holotype***, female (IZAS); label details are shown in Fig. 7f.

#### Other material examined.

One male and one female: China, Zhejiang, Lin’an, West Tianmushan, July 13, 2012, collected by Guanglin Xie (YZU); one female: China, Zhejiang, Lin’an, Qingliangfeng, May 22, 2012, collected by Guanglin Xie (YZU).

#### Distribution.

China (Zhejiang, Taiwan).

#### Remarks.

The redescription and comments about this species refer to Xie et al. (2022).

### ﻿ Xenicotela villiersi

Taxon classificationAnimaliaColeopteraCerambycidae

(Breuning, 1960) comb. nov.

A7632D02-6D7A-5569-82F1-7E4F54FD72A9

Fig. 8

Monochamus villiersi Breuning, 1960: 33; Breuning 1961: 370. Type locality: Tonkin (Hoa Binh), Vietnam.

#### Type material examined.

**Figure 8.**
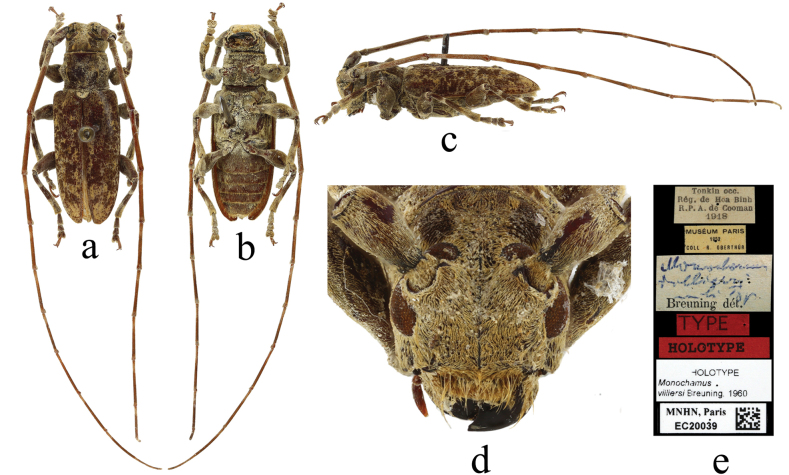
*Xenicotelavilliersi* (Breuning, 1960) comb. nov. **a** dorsal view **b** ventral view **c** lateral view **d** frontal view **e** labels.

***Holotype***, male (MNHN), the label details are shown in Fig. 8e.

#### Redescription.

**Male.** Body length 13.0 mm, humeral width 4.5 mm. Body mostly dull reddish brown, clothed with greyish-yellow to pale-yellow and brown pubescence mottled on dorsal surface. Head and pronotum slightly darker than elytra; legs somewhat blackish brown. Head decorated with a dark-brown pubescent patch behind each upper lobe of eyes and a pair of homogeneous patches located at the basal half of pronotum, edged with pale-yellow pubescence and widely separated anteriorly. Antennae reddish brown; scape and pedicel densely clothed with greyish-yellow pubescence; base and extreme apex of antennomeres III–X; base and apical half of antennomere XI annulated with greyish-yellow pubescence; basal five segments fringed with sparse greyish-yellow setae ventrally. Scutellum covered with whitish yellow throughout. Elytra dull reddish brown, mottled with greyish-yellow to pale-yellow and brown pubescence. Tibiae decorated with a narrow subbasal and a wide apical annulus of greyish-yellow to pale-yellow pubescence.

Frons transverse, densely and finely punctate; lower lobes of eyes about as long as genae. Antennae long, about 2.5 times as long as body, with the apex of the fifth segment or the base of the sixth one exceeding the elytral apex; scape robust and short, base narrowed, apex distinctly constricted before cicatrix; antennomere III slightly longer than antennomere IV, about 2.5 times as long as scape; extreme apex of antennomeres III–X obviously thickened inwards. Pronotum transverse; lateral spine short and blunt, coniform; disc dotted with fine punctures. Scutellum lingulate. Elytra elongate, about 2.2 times as long as width across humeri, with subparallel sides and rounded apices; surface finely punctate, the punctures gradually becoming finer and sparser towards apex; disc slightly raised at centre of basal third, followed by a weak central depression. Legs moderately long, femora slightly clavate, claws divaricate.

**Female.** Unknown.

#### Distribution.

Vietnam (Tonkin).

#### Comments.

This species has the mesotibiae without grooves near the apex, the head decorated with two black velvet patches behind the upper eye lobes, the scape distinctly constricted before the apical cicatrix, the basal five antennomeres fringed ventrally and antennomeres III–XI annulated with light-coloured pubescent rings on the base and apex, the pronotum with two black velvet patches on the base, the elytra mottled with light-coloured pubescence mixed with brown pubescence and the tibiae ringed with dark and light-coloured pubescence. All characters are consistent with the genus *Xenicotela*.

This species is very similar to *X.bimaculata*, from which it can be distinguished by the male antennae being much longer (about 2.5 times as long as body), the scutellum wholly covered with light-coloured pubescence, the base of the tibiae mostly dark with a light-coloured pubescent ring and the elytra more mottled. In *X.bimaculata*, the male antennae are only about 2.0 times as long as body, the scutellum is mostly clothed with light-coloured pubescence and the base of the tibiae is mostly clothed with light-coloured pubescence. *Xenicotelavilliersi* is also similar to *X.convexicollis* but differs in antennomeres V–VIII more protruding inwards at the apex and the elytra less elongate, without a transverse dark band behind the middle.

### ﻿ Xenicotela binigricollis

Taxon classificationAnimaliaColeopteraCerambycidae

(Breuning, 1965) comb. nov.

9FC1D09D-6447-516C-A8D9-90C2CB69FD3A

Fig. 7g, h

Monochamus binigricollis Breuning, 1965: 51; Rondon and Breuning 1970: 461; Lin and Tavakilian 2019: 310. Type locality: Pak Kading, Laos.

#### Type material examined.

***Holotype***, female (BPBM), Laos: Pak Kading, Paksane area, May, 1964, coll. J. A. Rondon.

#### Other material examined.

One female, Vietnam: Bac Kạn Province, National Park (LGBC).

#### Redescription.

**Female.** Body length 13.0 mm, humeral width 4.0 mm. Body mostly blackish brown, clothed with off-white, pale yellowish-brown to greyish-yellow and dark-brown pubescence. Head decorated with a dark-brown pubescent patch behind each upper eye lobe, a pair of homogeneous patches located at the base half of pronotum, widely separated anteriorly and indistinctly edged with pale-yellow pubescence on anterior and lateral sides. Antennae reddish brown; scape and pedicel densely clothed with greyish-yellow pubescence; base and extreme apex of antennomeres III–X; base and apical half of antennomere XI annulated with greyish-yellow pubescence; basal five segments fringed with sparse greyish-yellow setae ventrally. Scutellum completely clothed with greyish-yellow pubescence. Elytra dark brown, clothed with off-white, pale yellowish-brown to greyish-yellow and dark-brown pubescence forming a mottled pattern, distinctly dotted with irregular dark-brown spots; each elytron adorned with a large broad dark-brown median patch, slightly reduced near the suture. Tibiae decorated with a narrow subbasal and a wide apical annulus of greyish-yellow to pale-yellow pubescence.

Frons transverse, finely punctate; lower eye lobes slightly longer than genae. Antennae long, about 1.9 times as long as body; scape robust and short; base narrowed; apex distinctly constricted before cicatrix; antennomere III a little longer than antennomere IV, about 2.0 times as long as scape; antennomeres III–X slightly thickened at extreme apex. Pronotum transverse; lateral spine coniform, with pointed apex; disc dotted with fine punctures. Scutellum lingulate. Elytra elongate, about 2.3 times as long as width across humeri, with subparallel sides and rounded apices; punctures a little coarser and sparser than those on head and pronotum, sparser and even finer towards the apex; disc slightly raised at centre of basal third, followed by a weak central depression. Legs moderately long, femora slightly clavate, claws divaricate.

**Male.** Unknown.

#### Distribution.

Laos (Pak Kading), Vietnam (Tonkin).

#### Comments.

This species has the antennae with the scape robust and short and distinctly constricted before the cicatrix, basal five antennomeres fringed with sparse setae ventrally, and antennomeres III–XI annulated with light-coloured pubescence on base and apex; the pronotum with the lateral spine small, short and coniform, and the middle legs with the tibia without grooves. These characters are consistent with the genus *Xenicotela*.

This species is similar to *X.distincta* and *X.griseomaculata*, from which it differs in the lower eye lobe being longer than gena, the scutellum completely covered with light-coloured pubescence, and the base and the apex of elytra mostly clothed with light-coloured pubescence interspersed with small irregular dark-brown spots. In *X.distincta*, the lower eye lobe is about as long as gena, the scutellum is only clothed with the light-coloured pubescence on the edge and the elytra are not dotted with small irregular dark-brown spots. In *X.griseomaculata*, the lower eye lobe is shorter than gena, the scutellum is not clothed with light-coloured pubescence on the basal centre, and the base and apex of the elytra are mostly dark, adorned with light-coloured pubescent patches.

Wang (1998) incorrectly identified the specimens of *X.distincta* from Guizhou and Yunnan as this species on the basis of the main features of two black spots behind the upper eye lobes and at the base of pronotum respectively. In fact, *X.binigricollis* can be easy distinguished from *X.distincta*, as mentioned above. Consequently, it must be excluded from the fauna of China based on the information currently available.

### ﻿ Xenicotela griseomaculata

Taxon classificationAnimaliaColeopteraCerambycidae

Xie, Barclay & Chen, 2022

5A18EC82-3862-58D2-ACA7-DD0753BF6603

Fig. 9a, b, g

Xenicotela griseomaculata Xie et al, 2022: 152. Type locality: Chongqing (Wuxi County), China.

#### Type material examined.

***Holotype***, male, China: Chongqing, Wuxi County, Xiabao township, Shuanghe Village, 31°21'4"N, 109°11'24"E, July 26, 2019, coll. Bin Chen.

**Figure 9.**
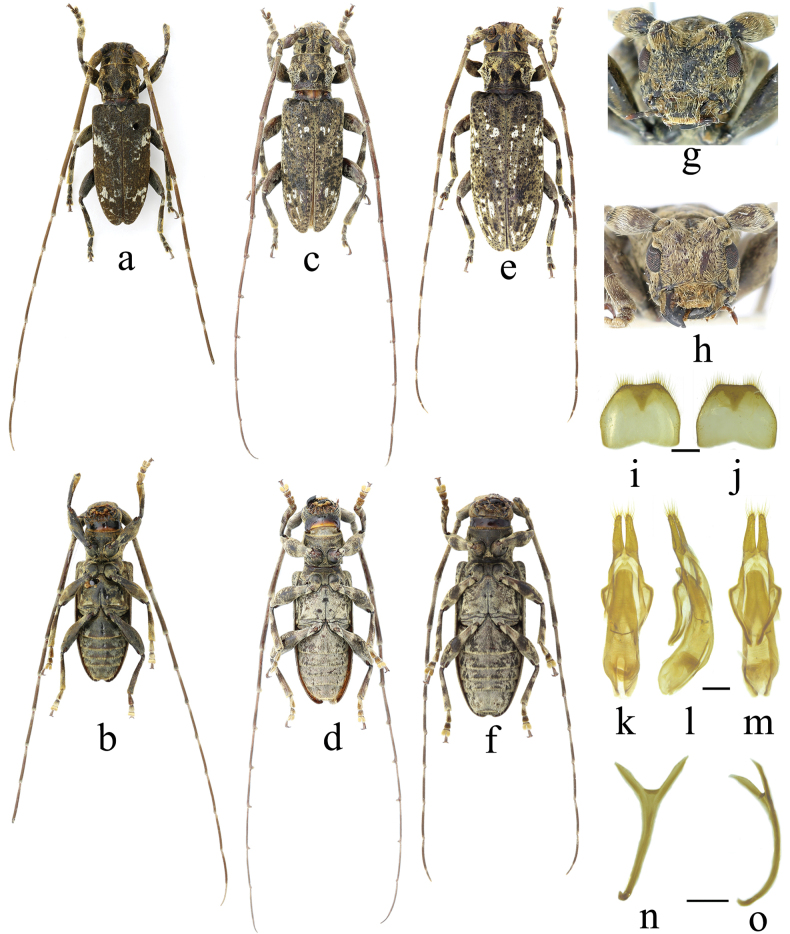
*Xenicotela* spp. **a, b, g** holotype of *Xenicotelagriseomaculata*, male **c–f, h–o***Xenicotelamucheni* sp. nov. **c, d, h** male **e, f** female **a, c, e, i, k** dorsal view **b, d, f, j, m, n** ventral view **l, o** lateral view **g, h** frontal view **i–o** male genitalia **i, j** tergite VIII **k–l** tegmen + median struts **n, o** spiculum gastrale. Scale: 0.5 mm.

#### Distribution.

China (Chongqing).

#### Remarks.

Description and comments on this species are provided by Xie et al. (2022).

### ﻿ Xenicotela mucheni sp. nov.

Taxon classificationAnimaliaColeopteraCerambycidae

B2F6A438-5F4F-510E-8603-89F22CC58AE3

https://zoobank.org/ED984BE9-2323-4C9A-923B-F31931DAC3B2

Fig. 9c–f, h–o

#### Type material.

***Holotype***: male, China: Yunnan Province, Ruili (瑞丽), July 29, 2018, coll. local collector, deposited in the Insect Collection, College of Agriculture, Yangtze University (YZU). ***Paratypes***: one male and two females, China: Yunnan Province, Yingjiang County (盈江), Xima (昔马), Alt. 600–1200 m, July 29 to August 10, 2018, coll. by local collector, deposited in the Collection of Mu Chen (MCC, Shanghai, China).

#### Description.

**Male**. Body length 16.2–19.0 mm, humeral width 5.1–5.7 mm. Body mostly dark brown; antennae and legs mostly dull reddish brown, clothed with greyish-yellow, greyish-white and black pubescence forming maculations. Head densely clothed with greyish-yellow pubescence, denser and longer on labrum and clypeus, with a long oval black velvet spot behind each upper eye lobe. Antennae clothed with greyish-yellow pubescence; base and extreme apex of antennomeres III–X, basal fifth and apical two-fifths of antennomere XI annulated with sparse greyish-yellow to greyish-white pubescence. Pronotum clothed with greyish-yellow pubescence on the middle and greyish-white pubescence at sides, decorated with a short finger-like black spot on each side of basal half with apex directed obliquely outwards, base broken by a patch of greyish-yellow pubescence on the middle. Scutellum clothed with greyish-yellow pubescence, thinly edged with more light-coloured pubescence. Elytra mostly clothed with greyish-yellow pubescence, interspersed with irregular dark-brown pubescent spots throughout and white spots mainly on basal and apical fourth. Underside clothed with denser pubescence, decorated with irregular dark-brown spots on both sides; ventrites fringed with greyish-yellow pubescence on the apical margin. Legs mostly clothed with greyish-yellow pubescence; tibiae decorated with a dark pubescent ring at middle.

Head finely and sparsely punctate; frons transverse, slightly convex, with a smooth longitudinal median sulcus extending to occiput. Eyes coarsely faceted; lower lobe about as long as gena. Antennae long, about 2.1–2.3 times as long as body; scape robust, thin at base, distinctly constricted before cicatrix; antennomere III distinctly longer than antennomere IV, about 2.7 times as long as scape; antennomere IV longer than antennomere V, antennomeres V–X strongly toothed inwards at apex. Pronotum transverse, anterior and posterior margins subequal in width; lateral spine coniform, with blunt apex; disc slightly convex, finely and sparsely punctate, with a little flat centre. Scutellum lingulate. Elytra elongate, about 2.3 times as long as width across humeri, with subparallel sides and rounded apices; surface punctures a little coarser than those on head and pronotum, gradually becoming finer and sparser towards apex, with basal fourth slightly longitudinally elevated centrally. Underside inconspicuously punctate; apical margin of distal ventrite nearly straight. Legs moderately long, femora slightly clavate, mesotibiae without grooves near external apex, claws divaricate.

***Male genitalia*.** Tergite VIII with both sides subparallel at basal third, then converging straight to apex, apex broadly truncated, clothed with short to medium straight setae along apical and lateral sides. Tegmen about 2.37 mm long, maximum width of ringed part about 0.97 mm. Paramere about 0.39 mm long, base about 0.25 mm wide, length/width ratio about 1.56, rounded apically, clothed with setae of different lengths and thicknesses at apex. Median lobe about as long as tegmen, slightly arcuate in lateral view, apical margin of dorsal plate and ventral plate rounded; median struts relatively broad, about one-half as long as median lobe.

**Female.** Body length 24.1–27.1 mm, humeral width 7.3–8.5 mm. Similar to male, antennae about 1.8 times as long as body; elytra about 2.2 times as long as width across humeri; antennomeres V–X slightly thickened apically.

#### Distribution.

China: Yunnan.

#### Etymology.

The new species is named after Mr Chen Mu, in gratitude for his offering the material of this new species for this study.

#### Comments.

The new species differs from other species of the genus in the elytra with distinct, small, separate, irregular, white spots and the male antennae with prominent teeth on the apices of antennomeres V–IX.

### ﻿Key to the known species of *Xenicotela* Bates

**Table:** 

1	Elytra mostly clothed with greyish-yellow pubescence, without whitish pubescent patches	**2**
–	Elytra decorated with distinct greyish white or white pubescent spots or, at least, with light-coloured pubescent maculations of off-white pubescence mixed with greyish-yellow pubescence	**6**
2	Pronotum with two distinct dark-brown spots	**3**
–	Pronotum without such spots; elytral pubescence thin; each elytron with an incomplete transverse dark-brown patch after middle	***Xenicotelapardalina* (Bates, 1884)**
3	Scutellum wholly covered with light-coloured pubescence	**4**
–	Scutellum only clothed with light-coloured pubescence on edge, with dark-brown pubescence on central part	**5**
4	Light-coloured pubescent ring at base of antennomere III relatively long, about one-fourth as long as antennomere III; dark-brown spots on pronotal base distinctly edged with pale-yellow pubescence; each elytron without distinct transverse dark patches	***Xenicotelavilliersi* (Breuning, 1960), comb. nov.**
–	Light-coloured pubescent ring on base of antennomere III shorter than one-fourth of length of antennomere III; dark-brown spots on pronotal base without conspicuous pubescent border; each elytron with a distinct but incomplete transverse dark patch	***Xenicotelaconvexicollis* (Gressitt, 1942)**
5	Each elytron with a distinct large transverse dark-brown patch on the middle	***Xenicoteladistincta* (Gahan, 1888)**
–	Each elytron with irregular dark-brown patches that do not fuse into a distinct large transverse dark-brown patch on middle	***Xenicotelabimaculata* (Pic, 1925)**
6	Apex of male antennomeres V–IX only weakly protruding inwards; elytra with relatively large light-coloured pubescent patches	**7**
–	Apex of male antennomeres V–IX strongly toothed inwards; elytra with scattered irregular small white pubescent spots	***Xenicotelamucheni* sp. nov.**
7	Apical fourth of elytra mostly clothed with light-coloured pubescence, with irregular dark spots	***Xenicotelabinigricollis* (Breuning, 1965), comb. nov.**
–	Apical fourth of elytra mostly dark, with greyish-white pubescent patches	***Xenicotelagriseomaculata* Xie, Barclay & Chen, 2022**

## ﻿Discussion

The species of the genus *Xenicotela* have a small body size (usually less than 20 mm in length) and are similar in appearance to the small-bodied species of the genus *Monochamus* and are often treated as members of the latter. The key distinguishing feature between these two genera is the presence or absence of an oblique groove near the apex of the mesotibia. In *Xenicotela*, the mesotibia lacks a groove near the apex. Furthermore, the scape is short, distinctly narrowed at the base, slightly swollen in the middle, clearly constricted before the apex, appearing subfusiform; the lateral spine of the pronotum is short and blunt, coniform. Antennomeres with light-coloured pubescent rings at the base and apex and fringes on the lower sides appear to be stable generic features in *Xenicotela*, while the presence or absence of fringes on the lower sides of the antennae cannot be used to distinguish these two genera, as several subgenera of *Monochamus* occurring in Africa, such as *Ethiopiochamus* Dillon & Dillon, 1961, *Parochamus* Dillon & Dillon, 1961 and *Quasiochamus* Dillon & Dillon, 1961, are all fringed on the lower edge of the antennae (Dillon and Dillon 1961).

**Figure 10.**
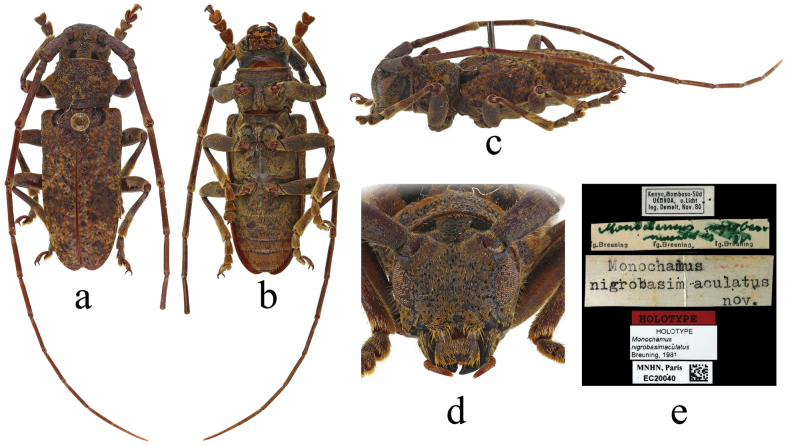
Holotype of *Monochamusnigrobasimaculatus* Breuning, 1981, Mombasa (Ukunda), Kenya **a** dorsal view **b** ventral view **c** lateral view **d** frontal view **e** labels.

An African species, Monochamus (Quasiochamus) nigrobasimaculatus Breuning, 1981 (Fig. 10), is very similar to members of *Xenicotela* in appearance, being of comparable body size, with two black velvet spots behind the upper eye lobes, the light-coloured pubescent rings at the base and apex on antennomeres III–XI, the light-coloured and dark pubescent rings on the femur, and the elytra mostly mottled, with two inconspicuous transverse dark spots after the middle. Although Breuning (1981) stated that there are no fringes on the lower sides of the antennae (non frangées en dessous), photographs of the holotype show that the antennae are distinctly fringed with the sparse pale-yellow setae below on basal five antennomeres, which also matches well with the features of *Xenicotela*. However, this species has large lateral pronotal spines and cylindrical scapes with unexpanded middle parts, which make it slightly different from *Xenicotela*. The presence or absence of a groove near the apex of the mesotibia is difficult to determine from the photographs and can only be determined after examination of the holotype. If the mesotibia has no groove, this species should be transferred into *Xenicotela*; conversely, if the mesotibia has a groove, its current status should be retained. As we were so far unable to directly examine the holotype, we are unable to draw a conclusion.

*Monochamus* is a large genus in the tribe Laminii, currently comprising 21 subgenera and over 160 species. It shows a considerable morphological variation among subgenera, which is well worth reviewing. In recent years, some species have been removed from *Monochamus* to other genera: *M.gravidus* Pascoe, 1858 to *Meges* Pascoe, 1866 (Bi et al. 2022), *M.serratus* Gahan, 1906, *M.semigranulatus* Pic, 1925, *M.asper* Breuning, 1935 and *M.latefasciatus* Breuning, 1944 to *Trachystohamus* Pic, 1936 (Vitali and Gouverneur 2022), *M.convexicollis* Gressitt, 1942 to *Xenicotela* (Xie et al. 2022), and *M.fruhstorferi* Breuning, 1964 to *Annamanum* Pic, 1925 [sub *Annamanumlunulatum* (Pic, 1934)] (Lin and Lingafelter 2018). In the present study, two additional species, *M.binigricollis* and *M.villiersi*, are transferred to *Xenicotela*. As the genera are studied in more depth it is likely that more species will change their current status.

## Supplementary Material

XML Treatment for Xenicotela

XML Treatment for Xenicotela pardalina

XML Treatment for Xenicotela distincta

XML Treatment for Xenicotela bimaculata

XML Treatment for Xenicotela convexicollis

XML Treatment for Xenicotela villiersi

XML Treatment for Xenicotela binigricollis

XML Treatment for Xenicotela griseomaculata

XML Treatment for Xenicotela mucheni

## ﻿Additional information

### Author ORCIDs

Guanglin Xie https://orcid.org/0000-0003-3273-8985

Maxwell V. L. Barclay https://orcid.org/0000-0003-4989-2014

Wenkai Wang https://orcid.org/0000-0002-4482-076X

### Data availability

All of the data that support the findings of this study are available in the main text.
